# Use what you have: leveraging microbiology support to develop a cumulative antibiotic susceptibility report for antimicrobial stewardship at a district hospital in Ghana

**DOI:** 10.1093/jacamr/dlae129

**Published:** 2024-08-21

**Authors:** Benedicta Bosu, Obed Kwabena Offe Amponsah, Phyllis Tawiah, Eric Darko, Nana Akua Abruquah, Annabella Bensusan Osafo, Emmanuel Sarkodie, Nana Bugyei Buabeng, Otridah Kapona, Alex Owusu-Ofori, Kwame Ohene Buabeng, Nana Kwame Ayisi-Boateng

**Affiliations:** University Hospital, Kwame Nkrumah University of Science and Technology (KNUST), Kumasi, Ghana; Department of Pharmacy Practice, Faculty of Pharmacy and Pharmaceutical Sciences, KNUST, Kumasi, Ghana; University Hospital, Kwame Nkrumah University of Science and Technology (KNUST), Kumasi, Ghana; Department of Medicine, School of Medicine and Dentistry, KNUST, Kumasi, Ghana; University Hospital, Kwame Nkrumah University of Science and Technology (KNUST), Kumasi, Ghana; University Hospital, Kwame Nkrumah University of Science and Technology (KNUST), Kumasi, Ghana; University Hospital, Kwame Nkrumah University of Science and Technology (KNUST), Kumasi, Ghana; University Hospital, Kwame Nkrumah University of Science and Technology (KNUST), Kumasi, Ghana; University Hospital, Kwame Nkrumah University of Science and Technology (KNUST), Kumasi, Ghana; Zambia National Public Health Institute, Lusaka, Zambia; Department of Clinical Microbiology, School of Medicine and Dentistry, KNUST, Kumasi, Ghana; School of Pharmacy, University of Health and Allied Sciences, Ho, Ghana; University Hospital, Kwame Nkrumah University of Science and Technology (KNUST), Kumasi, Ghana; Department of Medicine, School of Medicine and Dentistry, KNUST, Kumasi, Ghana

## Abstract

**Background:**

Antibiograms provide effective support for empirical prescribing and antimicrobial stewardship programmes (ASPs). In low-resource settings, microbiology systems to develop antibiograms may be rudimentary or entirely lacking, which may place such facilities at a disadvantage. Notwithstanding this, facilities should use what they have to support ASPs to inform evidence-based antibiotic use. We report how an antibiogram was developed at a district hospital in Ghana to support its ASP.

**Methods:**

This was a retrospective analysis of antibiotic susceptibility testing (AST) results from the University Hospital, KNUST from January to December 2021. Data were exported from the hospital’s laboratory information system to Microsoft Excel (Version 2013). IBM SPSS Statistics (Version 25) and Epi Info™ Version 7 were used for statistical analyses.

**Results:**

Overall, 1949 cultures were performed, 392 (20.1%) growing bacterial pathogens. Per the CLSI M39-A4 standard guidelines for antibiograms, only 360 of the bacterial isolates were used for the analyses. The majority of isolates were from urine (187; 51.9%). Among the Gram-negative bacteria, there was low susceptibility to amoxicillin/clavulanic acid (28%), cephalosporins (11%–35%) and meropenem (21%), but high susceptibility to amikacin (96%) and levofloxacin (81%). Low susceptibility of Gram-positive isolates to amoxicillin/clavulanic acid (34%), meropenem (34%) and penicillins (27%–35%) was also recorded, but high susceptibility to ciprofloxacin (80%), gentamicin (79%) and vancomycin (76%).

**Conclusion:**

High levels of bacterial resistance to cephalosporins and meropenem in the antibiogram were reported. This antibiogram highlighted the urgent need for pragmatic steps to curb antibiotic resistance through ASPs using strategies that positively improve clinicians’ knowledge and prescribing practices.

## Introduction

The spate of antimicrobial resistance (AMR) and its spread globally, with associated morbidity and mortality,^1^ as well as limited treatment options for infections, is a significant threat to health and economic development.^2,3^ Annually, nearly 700 000 deaths are attributed to AMR and it is predicted to rise to 10 million deaths per annum by 2050.^4^ A *Lancet* publication estimated that in 2019, 4.95 million deaths were associated with bacterial resistance globally, of which 1.27 million deaths were directly attributable to resistance.^5^ Low-resource countries bear the brunt of this global AMR threat.^6^ According to the 2020 and 2021 reports by the Global Antimicrobial Resistance and Use Surveillance System (GLASS), the rate of infections with AMR pathogens is higher in low- and middle-income countries (LMICs) such as Ghana compared with high-income countries (HICs).^7,8^ Several studies in Ghana have assessed AMR trends among pathogens from cultured specimens and reported high rates (>70%) of resistance.^9–11^

Factors such as lack of evidence-based treatment guidelines, inadequate laboratory capacity, and blind treatment of infections by physicians have limited effective AMR surveillance in LMICs.^12–14^ The 2021 WHO GLASS report indicated that out of the 12 countries that submitted no record of their national AMR surveillance, 8 (67%) were LMICs.^8^ This warrants a call to strengthen clinical microbiology laboratories in LMICs to boost their capacity for surveillance of AMR and antimicrobial use.^15^

In Ghana, a recent point prevalence survey (PPS) across the nation reported that one in every two patients who visit a hospital is likely to receive empirical antibiotic treatment that is not informed by an antibiogram.^16^ Cumulative antibiotic susceptibility reports (antibiograms) are therefore useful in the development of local antimicrobial prescribing guidelines, minimizing the inappropriate use of broad-spectrum antibiotics, monitoring resistance trends and enhancing patient safety.^17–19^

Developing antibiograms requires microbiology capacity in the form of equipment, reagents and consumables, as well as skilled personnel, who are often inadequate or completely lacking in LMICs. Notwithstanding this, all health facilities stand to benefit from antibiograms and would be best served leveraging their available resources to produce and use them. We report on the process for developing an antibiogram in the University Hospital of Kwame Nkrumah University of Science and Technology (KNUST) in Kumasi, Ghana using retrospective antibiotic susceptibility data of patients to support the hospital’s antimicrobial stewardship programme. Additionally, most AMR surveillance studies in Ghana have been conducted in tertiary and secondary healthcare facilities, with very few done in primary care facilities and none reported from the Ashanti region, the second largest administrative region.^9–11,16,20–25^ This manuscript will help to bridge this data gap on AMR surveillance in Ghana. This report details valuable information on local resistance patterns identified that were used to improve antimicrobial stewardship and subsequently to develop antibiotic use guidelines in the hospital.

## Methods

### Study design

We conducted a retrospective evaluation of culture and antibiotic susceptibility data of patients attending KNUST, Kumasi, Ghana, from 1 January to 31 December 2021. The study setting has been previously described by Amponsah *et al*.^26^

### Microbiology services

The hospital has a central laboratory where all patient samples collected are sent for all the required analyses. The microbiology unit occupies a space within this laboratory, has been functional for the past 10 years and can perform bacteriological cultures of all body fluids except stool and sputum, some fungal cultures, and parasitological investigations. Due to limited resources, most of its investigations are done manually. However, the lab is run by well-qualified staff who are able to utilize the available resources with internal quality assurance systems to achieve desired results to support antimicrobial stewardship at the hospital.

### Data collection

Patient/study data were retrieved from the hospital’s laboratory information system (LIS). This is part of the hospital’s electronic medical records (EMR), which is largely a local area network (LAN)-based system with some components being web-based. Clinical information regarding the patient is updated on the EMR by the physician/physician assistant at the time of consultation and laboratory requests are automatically sent to the LIS for processing and reporting.

### Specimen collection, transport and processing

Urine, blood, CSF, ascitic and synovial fluids, high vaginal swabs (HVSs) and swabs from the endocervix, wounds, urethra, throat, nasopharynx, eyes and ears were received for bacterial culture. Sputum and stool cultures are outsourced to external laboratories and hence no data on them were reported by the laboratory. Specimens that yielded bacterial pathogens were included in the study. By the CLSI M39-A4 standard guidelines,^27^ only the first isolate per patient within the period analysed was used in the antibiogram, irrespective of the specimen type. Culture and identification of bacterial isolates from specimens were all performed using conventional microbiological techniques. All specimens were transported to the laboratory at room temperature and processed within 30 min after collection. Urine specimens were inoculated on cystine-lactose-electrolyte-deficient (CLED) agar; HVSs and urethral swabs were inoculated on blood and chocolate agars, but the inoculation of all other samples was done on blood, chocolate and MacConkey agars. All CLED, MacConkey and blood agar plates were incubated aerobically at 35–37°C for 24 h, while chocolate agar plates were kept in a carbon dioxide jar at 35–37°C for 24–48 h.

The conventional techniques used for identification involved: Gram staining, colonial morphology, biochemical tests such as catalase test, coagulase test, oxidase test, triple sugar iron (TSI) test, urease test, citrate test, indole test and disc tests such as optochin disc test, bacitracin disc test and novobiocin disc test.

### Antibiotic susceptibility testing (AST)

The AST was performed using Kirby–Bauer’s disc diffusion technique and interpreted as per the CLSI guidelines.^28^ The AST panel included penicillin (1.5 μg), ampicillin (10 μg), cloxacillin (5 μg), amikacin (30 μg), gentamicin (10 μg), cefuroxime (15 μg), cefotaxime (30 μg), ceftriaxone (30 μg), ceftazidime (20 μg), amoxicillin/clavulanic acid (30 μg), piperacillin (20 μg), piperacillin/tazobactam (100/10 μg), ofloxacin (5 μg), ciprofloxacin (5 μg), levofloxacin (5 μg), norfloxacin (20 μg), co-trimoxazole (25 μg), meropenem (10 μg), nitrofurantoin (300 μg), nalidixic acid (30 μg), chloramphenicol (30 μg), vancomycin (30 μg) and erythromycin (5 μg).

### Ethics

As this study reports on routine laboratory work using routinely collected data with no patient identifiers, no additional approvals were needed. However, the antimicrobial stewardship programme received institutional consent from the hospital management and ethical approval from the Committee on Human Research Publications and Ethics (CHRPE) of KNUST, Kumasi, Ghana (CHRPE/AP/470/22).

### Data analysis

Data were exported from the hospital’s LIS into Microsoft Excel (Version 2013). IBM SPSS Statistics Version 25 (IBM Corp; 2017, Armonk, NY, USA) and Epi Info™ Version 7 (CDC, Atlanta, GA, USA) were used for the statistical analyses. Descriptive analysis was performed on sociodemographic, pathogenic and antibiotic profile data, with results presented in tables as frequencies and percentages. The data were not normally distributed hence IQR age was reported.

## Results

A total of 1949 patient specimens were cultured, comprising 807 urines (41.41%), 683 HVSs (35.04%), 168 blood specimens (8.62%), 163 urethral swabs (8.36%), 78 wound swabs (4.00%), 20 CSFs (1.03%) and 30 others (1.54%). Seven hundred and fifty-three isolates were obtained, of which only 392 (52.1%) were bacterial. As per the CLSI M39-A4 standard guidelines for antibiograms,^27^ only 360 of the bacterial isolates, each of which represented an isolate from a unique individual patient (Table 1), were used for the analyses [Table 2; Figure S1 (available as Supplementary data at *JAC-AMR* Online)].

**Table 1.** Demographic characteristics of patients with bacterial infections in 2021 at the University Hospital, KNUST

Variable	Frequency (*n* = 360)	Percentage (%)
Age category (years)		
0–14	15	4.2
15–29	173	48.1
30–44	69	19.2
45–59	41	11.4
≥60	62	17.2
Gender		
Male	114	31.7
Female	246	68.3
Department		
Children’s ward	3	0.8
Emergency room	5	1.4
Female’s ward	27	7.5
Male’s ward	16	4.4
Special ward	6	1.7
Outpatient department	303	84.2
Provisional diagnosis		
Indicated	124	34.4
Not indicated	236	65.6

**Table 2.** Distribution of bacterial isolates amongst clinical specimens cultured at KNUST Hospital

Specimen	Gram-negative bacteria									Gram-positive bacteria				
	Enterobacteriaceae (non-speciated)	*Klebsiella* spp.	*E. coli*	*Pseudomona*s spp.	*Proteus* spp.	*Salmonella* spp.	*Enterobacter* spp.	*Neisseria gonorrhoeae*	Non-lactose-fermenting rods	*S. aureus*	*Streptococcus pneumoniae*	*Streptococcus* spp.	Branching filamentous rods	Total (%)
Urine	115	26	20	—	—	1	—	—	—	24	—	—	1	187 (51.9%)
HVS	8	1	8	—	—	—	—	—	—	26	—	12	2	57 (15.8%)
Blood	1	3	3	20	—	6	3	—	1	3	5	1	—	46 (12.8%)
Wound	12	3	1	8	3	—	—	—	1	16	—	—	—	44 (12.2%)
Urethral swab	1	—	—	—	—	—	—	1	—	16	—	2	—	20 (5.6)
Ear swab	1	—	—	1	—	—	—	—	—	2	—	—	—	4 (1.1)
Eye swab	—	—	—	—	—	—	—	—	—	—	—	1	—	1 (0.3)
Abscess	—	—	—	—	—	—	—	—	—	1	—	—	—	1 (0.3)
Total (%)	138 (38.3)	33 (9.2)	32 (8.9)	29 (8.1)	3 (0.8)	7 (1.9)	3 (0.8)	1 (0.3)	2 (0.6)	88 (24.4)	5 (1.4)	16 (4.4)	3 (0.8)	360 (100)

Of the patients whose samples were submitted to the laboratory, 65.6% lacked a clinical diagnosis at the time of submission of the sample, while 34.4% had a provisional diagnosis, with the majority (94.9%) of the samples coming from outpatients. Urinary tract infection (UTI) was the most common diagnosis (Table S1), with isolates sensitive to amikacin and levofloxacin (Figure S2).

### Bacterial isolates: profile and distribution among specimens

Specimens that yielded isolates included urines (187/807; 23.2%), HVSs (57/683; 8.3%), blood (46/168; 27.4%), wound swabs (44/78; 56.4%), urethral swabs (20/163; 12.3%), ear swabs (4/6; 66.7%), eye swabs (1/4; 25%) and abscess specimens (1/5; 20%) (Figure S1). The major isolates obtained were non-speciated Enterobacteriaceae (138; 38.3%), *Staphylococcus aureus* (88; 24.4%), *Klebsiella* spp. (33; 9.2%) and *Escherichia coli* (32; 8.9%) (Figure S1).

### Antimicrobial susceptibility profile of isolates

#### Gram-negative isolates

**Figure 1.**
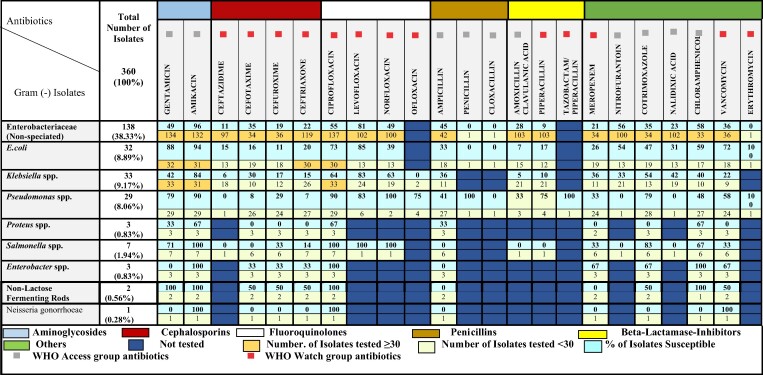
Susceptibility of Gram-negative isolates to selected antibiotics at KNUST Hospital.

All the Gram-negative bacterial species that met the threshold of greater than 30 isolates, per the CLSI antibiogram reporting guidelines,^27^ belonged to the Enterobacteriaceae family, comprising *E. coli*, *Klebsiella* spp. and other Enterobacteriaceae (non-speciated). Of those that met the CLSI threshold, there was generally low susceptibility to commonly used antibiotics like β-lactamase inhibitors (amoxicillin/clavulanic acid 28%, piperacillin/tazobactam 9%), cephalosporins (ceftazidime 11%, cefuroxime 19%, ceftriaxone 22%, cefotaxime 35%), penicillins (ampicillin 45%), nalidixic acid (23%), meropenem (21%), co-trimoxazole (35%) and vancomycin (36%). High susceptibility to amikacin (84%–96%) and levofloxacin (81%) was evident amongst all the Gram-negative isolates. Although fewer than 30 isolates of *Pseudomonas* spp., *Salmonella* spp., *Enterobacter* spp. and *Proteus* spp. were recorded, a low susceptibility trend was also observed for cephalosporins amongst them (Figure 1).

#### Gram-positive isolates

**Figure 2.**
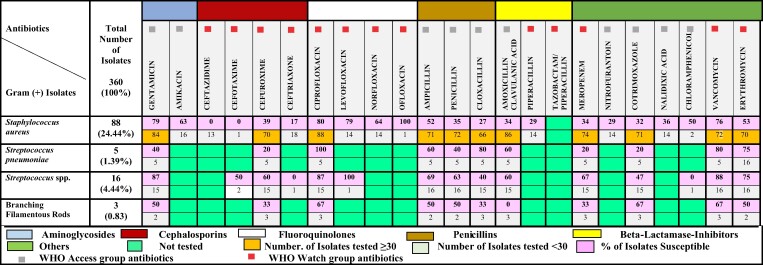
Susceptibility of Gram-positive isolates to selected antibiotics at KNUST Hospital.

*S. aureus* comprised almost 80% (78.6%) of the Gram-positive bacteria isolated, followed by *Streptococcus* spp. (18.8%) and branching filamentous rods (2.7%). Only *S. aureus* met the CLSI threshold of >30 isolates per species. Again, there was grossly low susceptibility to amoxicillin/clavulanic acid (34%), meropenem (34%), co-trimoxazole (32%) and the penicillins (cloxacillin 27%, penicillin 35%). There was high susceptibility to gentamicin (79%), ciprofloxacin (80%) and vancomycin (76%). Again, though <30 isolates of *Streptococcus* spp. were recorded, similarly low susceptibility to cloxacillin was observed (Figure 2). There was high resistance of all the isolates to meropenem, co-trimoxazole, cephalosporins and amoxicillin/clavulanic acid (Figure 2).

## Discussion

Our data revealed that urine samples constituted the majority of the specimens cultured, consistent with a similar study from Ethiopia,^29^ where urine accounted for 57.8% of the samples cultured. Similar studies conducted in Ghana also reported that urine samples constituted a large proportion of all the specimens cultured, though their percentage was slightly lower.^30^ In this study, this may be because UTI was the most frequently suspected clinical diagnosis, and the majority of specimens were from female patients, who are at a higher risk of UTI than male patients.^31^ Generally, utilization of blood cultures is low in most health institutions in Ghana.^9,10^ At the University Hospital, KNUST, out of all the laboratory requests received for the year 2021, blood culture requests comprised less than 10% (8.6%), of which pathogens were recovered in 48 (28.6%). This may be because blood cultures are generally expensive and most patients are unable to afford it. Inadequate availability of culture bottles prior to initiation of antibiotics could also potentially be a factor limiting the number of culture samples that are sent for analyses. Additionally, the hospital is a district-level facility, which may not receive the most serious cases requiring investigations like blood and CSF cultures.

The majority of the isolates were Gram-negative bacteria (68.9%), which is similar to a Kenyan study reporting 67% for Gram-negatives.^32^ Similar findings have been reported in Ghana,^9,22^ India,^18^ Ethiopia^29^ and Zambia.^33^ The low susceptibilities to different antibiotic classes may suggest that most of the enterobacteria isolated may be ESBL producers. This poses a great threat because of limited treatment options. Several studies from Ghana,^9–11,22,23,34,35^ and other countries like India,^18^ Ethiopia,^29^ East Africa,^32^ Zambia^33^ and the USA^36^ have reported similarly low susceptibilities. Investment in advanced microbiology equipment for automation, species identification and timely reporting would be beneficial to fully identify such organisms and their resistance profiles to complement global surveillance.

There was low susceptibility of Gram-negative bacteria to meropenem (21%). Although some studies in Ghana^37–39^ have reported emerging meropenem resistance, the prevalence of resistance reported was relatively low compared with this study. In the case of the study by Bediako-Bowman *et al*.,^37^ meropenem resistance was mainly found in *E. coli* (1 of 139 isolates; 0.7%), *Pseudomonas* spp. (15 of 49 isolates; 31%) and *Acinetobacter baumannii* (6 of 23 isolates; 26%). In Nigeria, a study by Olu-Taiwo *et al*.^38^ recorded 59.8% meropenem-resistant *Acinetobacter*. However, most studies conducted in Ghana^9–11,23,34^ have reported >85% susceptibility of Gram-negatives to meropenem. These varying results may be due to differences in study setting, sample size, study population, prescription pattern, antibiotic therapy and epidemiology of the disease-causing organisms. Additionally, clinicians at the hospital may be using it predominantly for more severe infections after other therapies have failed, accounting for the high resistance. This highlights the importance of using local data as evidence for optimal antibiotic use.

The Gram-negative isolates were most susceptible (84%–96%) to amikacin and this is consistent with numerous studies in Ghana^9–11,22,23,34,35^ and elsewhere.^32,33,36,40^ This may suggest that the susceptibility to amikacin has not changed significantly over the years. Perhaps the low use of amikacin in KNUST hospital and other facilities in Ghana, resulting in lower exposure of microbes to this antibiotic, may account for its relatively high effectiveness against most isolates. Therefore, the efficacy of amikacin must be preserved by ensuring that policies are put in place to regulate its use extensively.

Amongst the Gram-positive bacteria, *S. aureus* was the only isolate that met the CLSI threshold of >30 isolates of a species and also exhibited low susceptibility to cefuroxime (39%), penicillins (27%–52%), amoxicillin/clavulanic acid (34%), co-trimoxazole (32%) and meropenem (34%), but was quite susceptible to vancomycin (76%), gentamicin (79%) and ciprofloxacin (80%). These outcomes are consistent with other research work done in Ghana^10,22,35^ and overseas.^15,29,32,33,41^ Another study from Saudi Arabia reported slightly higher susceptibility proportions for penicillins (41.3%–82.9%), co-trimoxazole (87.7%), vancomycin (98.7%) and ciprofloxacin (83.1%).^40^ Similarly, high susceptibility of *S. aureus* to vancomycin (86.4%), gentamicin (90.9%) and ciprofloxacin (95.5%) were reported in a study by Alshamahi *et al*., in Yemen.^42^ Unfortunately, MRSA screening was not done, but the poor susceptibility to cloxacillin (27%) observed indicates the need to screen for and confirm MRSA.

Based on the results obtained from this antibiogram, the antimicrobial stewardship committee of the hospital was able to develop a draft antibiotic use guideline, which has been reviewed and accepted by management, and is currently in use. The antimicrobial stewardship programme had hitherto used only persuasive initiatives but this guideline includes restrictive prescribing to optimize antibiotic use. Additionally, approval has been obtained to improve the capacity of the microbiology unit by the hospital management based on the output and feedback provided to them from this report. This includes a plan to acquire automated analysers for blood cultures, bacterial identification and AST to reduce sample turnaround times and contamination, and improve the overall efficiency of the laboratory. Health facilities in other low-resource settings would benefit from leveraging what they already possess to support the optimal use of antibiotics to ensure effective contributions to the global fight against AMR.

The data from this study will be shared with the Ghana national AMR coordinating committee, as well as with the WHO Ghana country office, to improve our knowledge of antibiotic resistance patterns in the country, bridge the data gap and inform the implementation of better infection control practices.

## Limitations

As a result of logistical limitations, not all the specific genera and species of bacterial isolates could be identified and were thus classified as Enterobacteriaceae (non-speciated).

The study did not distinguish between organisms from hospital-acquired infections (HAIs) and community-acquired infections (CAIs). Comparing the susceptibility patterns of organisms from HAIs and CAIs is planned for subsequent analysis, which would be very useful for directing antimicrobial stewardship and improvement of infection prevention and control in the hospital.

### Conclusions

In descending order, amikacin, levofloxacin and gentamicin were the antibiotics that the Gram-negative bacteria were susceptible to, while the Gram-positive bacteria were mostly susceptible to ciprofloxacin, gentamicin and vancomycin. High resistance of Gram-negative isolates to meropenem, and increased resistance of all isolates to the cephalosporins (ceftazidime, cefuroxime, cefotaxime, ceftriaxone), amoxicillin/clavulanic acid and other antibiotics are of concern. This information has been useful in guiding prescribing in the hospital and antibiotic use. Other health institutions will likely benefit from developing antibiograms to guide clinicians in the selection of antibiotics for empirical therapy of life-threatening infections whilst waiting for test results of culture for definitive therapy.

## Supplementary Material

dlae129_Supplementary_Data

## Supplementary data

Figures 1 and 2 and Table 1 are available as Supplementary data at *JAC-AMR* Online.
