# Supplementary material for: Use what you have: leveraging microbiology support to develop a cumulative antibiotic susceptibility report for antimicrobial stewardship at a district hospital in Ghana
Source: JAC Antimicrob Resist. 2024 Aug 21;6(4):dlae129. doi: 10.1093/jacamr/dlae129 (PMC11337119; doi:10.1093/jacamr/dlae129)
Supplement: dlae129_Supplementary_Data [file dlae129_supplementary_data.docx]

SUPPLEMENTARY FIGURES AND TABLES

**Supplementary figure 1: General Overview of Cultures Performed from 01/01/2021- 31/12/2021 in the University Hospital, KNUST**

| SPECIMEN | NO PATHOGENIC GROWTHS | PATHOGENIC GROWTHS | | | TOTAL  SPECIMENS RECEIVED | PATHOGENIC ISOLATES | | | BACTERIAL ISOLATES USED FOR ANTIBIOGRAM ANALYSES |
| --- | --- | --- | --- | --- | --- | --- | --- | --- | --- |
|  |  | SINGLE COLONIES | MIXED COLONIES | TOTAL PATHOGENIC GROWTHS |  | BACTERIAL  ISOLATES | FUNGAL  ISOLATES | TOTAL |  |
| URINE | 595 | 204 | 8 | 212 | 807 | 204 | 16 | 220 | 187 |
| HIGH VAGINAL SWAB (HVS) | 296 | 374 | 13 | 387 | 683 | 61 | 339 | 400 | 57 |
| BLOOD | 120 | 48 | 0 | 48 | 168 | 48 | 0 | 48 | 46 |
| URETHRAL SWAB | 141 | 22 | 0 | 22 | 163 | 20 | 2 | 22 | 20 |
| WOUND SWAB | 26 | 50 | 2 | 52 | 78 | 53 | 1 | 54 | 44 |
| CEREBROSPINAL FLUID (CSF) | 20 | 0 | 0 | 0 | 20 | 0 | 0 | 0 | 0 |
| ENDOCERVICAL SWAB (ECS) | 6 | 2 | 0 | 2 | 8 | 0 | 2 | 2 | 0 |
| EAR SWAB | 1 | 5 | 0 | 5 | 6 | 4 | 1 | 5 | 4 |
| ABSCESS | 4 | 1 | 0 | 1 | 5 | 1 | 0 | 1 | 1 |
| EYE SWAB | 3 | 1 | 0 | 1 | 4 | 1 | 0 | 1 | 1 |
| SYNOVIAL FLUID | 4 | 0 | 0 | 0 | 4 | 0 | 0 | 0 | 0 |
| ASCITIC FLUID | 1 | 0 | 0 | 0 | 1 | 0 | 0 | 0 | 0 |
| NASOPHARYNGEAL SWAB | 1 | 0 | 0 | 0 | 1 | 0 | 0 | 0 | 0 |
| THROAT SWAB | 1 | 0 | 0 | 0 | 1 | 0 | 0 | 0 | 0 |
| TOTAL | **1219**  **(62.5 %)** | 707 (96.8%) | 23 (3.2%) | **730**  **(37.5%)** | **1949**  **(100%)** | **392**  **(52.1%)** | **361**  **(47.9%)** | **753**  **(100%)** | **360**  **(100%)** |
|  |  | TOTAL = 730 (100%) | |  |  |  |  |  |  |

| Diagnosis | Frequency | Percentage |
| --- | --- | --- |
| Urinary Tract Infections | 53 | 42.75 |
| Genital Infections/ Sexually Transmitted Infections | 48 | 38.71 |
| Skin/ Soft Tissue Infections | 3 | 2.42 |
| Respiratory Infections | 3 | 2.42 |
| Blood stream Infections | 1 | 0.81 |
| Others | 16 | 12.90 |
| TOTAL | **124** | **100%** |

#

# **Supplementary Table 1: Clinical Diagnoses of Patients at the Time of Sample collection at the University Hospital, KNUST**

| **DIAGNOSIS** | **TOTAL**  **NUMBER**  **(124)** | ANTIBIOTICS | | | | | | | | | | | | | | | | | | | | | | |
| --- | --- | --- | --- | --- | --- | --- | --- | --- | --- | --- | --- | --- | --- | --- | --- | --- | --- | --- | --- | --- | --- | --- | --- | --- |
|  |  | **GENTAMICIN** | **AMIKACIN** | **CEFTAZIDIME** | **CEFOTAXIME** | **CEFUROXIME** | **CEFTRIAXONE** | **CIPROFLOXACIN** | **LEVOFLOXACIN** | **NORFLOXACIN** | **OFLOXACIN** | **AMPICILLIN** | **PENICILLIN** | **CLOXACILLIN** | **AMOXICILLIN CLAVULANIC ACID** | **PIPERACILLIN** | **TAZOBACTAM/**  **PIPERACILLIN** | **MEROPENEM** | **NITROFURANTOIN** | **COTRIMOXAZOLE** | **NALIDIXIC ACID** | **CHLORAMPHENICOL** | **VANCOMYCIN** | **ERYTHROMYCIN** |
| **URINARY TRACT INFECTIONS** | **53** | 57 | **95** | 16 | 27 | 41 | 24 | **72** | **89** | 63 | 0 | 67 | 57 | 57 | 25 | 9 | 0 | 28 | 65 | 44 | 29 | **70** | 50 | 57 |
|  |  | 49 | 43 | 32 | 1 | 17 | 41 | 53 | 35 | 33 | 0 | 21 | 7 | 7 | 40 | 35 | 0 | 18 | 35 | 18 | 35 | 10 | 18 | 7 |
| **GENITAL INFECTIONS** | **48** | **83** | 36 | 20 | 20 | 32 | 11 | **79** | 90 | 60 | 0 | 58 | 32 | 32 | 34 | 10 | 0 | 24 | 70 | 30 | 40 | 46 | **81** | 54 |
|  |  | 48 | 21 | 10 | 10 | 37 | 19 | 48 | 10 | 10 | 0 | 38 | 28 | 28 | 38 | 10 | 0 | 35 | 10 | 37 | 10 | 11 | 37 | 28 |
| **RESPIRATORY TRACT INFECTIONS** | **3** | 67 | 67 | 0 | 0 | 0 | 50 | 100 | 100 | 100 | 0 | 0 | 0 | 0 | 0 | 0 | 0 | 100 | 0 | 0 | 67 | 0 | 0 | 0 |
|  |  | 3 | 3 | 3 | 0 | 0 | 2 | 3 | 2 | 3 | 0 | 0 | 0 | 1 | 3 | 3 | 0 | 1 | 3 | 0 | 3 | 0 | 0 | 0 |
| **SKIN AND SOFT TISSUE INFECTIONS** | **3** | 67 | 100 | 0 | 50 | 67 | 0 | 100 | 0 | 0 | 0 | 100 | 100 | 0 | 100 | 0 | 0 | 67 | 0 | 67 | 0 | 50 | 100 | 100 |
|  |  | 3 | 2 | 0 | 2 | 3 | 1 | 3 | 0 | 0 | 0 | 3 | 1 | 1 | 1 | 0 | 0 | 3 | 0 | 3 | 0 | 2 | 3 | 1 |
| **BLOOD STREAM INFECTIONS** | **1** | 100 | 100 | 0 | 0 | 0 | 0 | 100 | 0 | 0 | 0 | 0 | 0 | 0 | 0 | 0 | 0 | 0 | 0 | 100 | 0 | 100 | 0 | 0 |
|  |  | 1 | 1 | 0 | 1 | 1 | 1 | 1 | 0 | 0 | 0 | 1 | 0 | 0 | 0 | 0 | 0 | 1 | 0 | 1 | 0 | 1 | 1 | 0 |
| **OTHERS** | **16** | 38 | 100 | 0 | 0 | 43 | 7 | 69 | 88 | 59 | 0 | 43 | 67 | 33 | 17 | 11 | 0 | 57 | 67 | 71 | 22 | 50 | 67 | 83 |
|  |  | 16 | 11 | 9 | 4 | 7 | 14 | 16 | 8 | 9 | 0 | 7 | 3 | 3 | 12 | 9 | 0 | 7 | 9 | 7 | 9 | 4 | 6 | 3 |
| Number of isolates tested %Susceptible Susceptibility >70% for more than 30 tested Isolates. | | | | | | | | | | | | | | | | | | | | | | | | |

**Supplementary Figure 2: Distribution of Antibiotic Susceptibility Pattern According to Clinical Diagnosis**
